# Nanofibrous Online Solid-Phase Extraction Coupled with Liquid Chromatography for the Determination of Neonicotinoid Pesticides in River Waters

**DOI:** 10.3390/membranes12070648

**Published:** 2022-06-24

**Authors:** Ivana H. Šrámková, Burkhard Horstkotte, Laura Carbonell-Rozas, Jakub Erben, Jiří Chvojka, Francisco J. Lara, Ana M. García-Campaña, Dalibor Šatínský

**Affiliations:** 1 Department of Analytical Chemistry, Faculty of Pharmacy in Hradec Králové, Charles University, Heyrovského 1203, 500 05 Hradec Králové, Czech Republic; horstkob@faf.cuni.cz (B.H.); satinsky@faf.cuni.cz (D.Š.); 2 Department of Analytical Chemistry, Faculty of Sciences, University of Granada, Av. Fuente Nueva s/n, E-18071 Granada, Spain; rozas@ugr.es (L.C.-R.); frjlara@ugr.es (F.J.L.); amgarcia@ugr.es (A.M.G.-C.); 3 Department of Nonwovens and Nanofibrous Materials, Faculty of Textile Engineering, Technical University of Liberec, Studentská 2, 461 17 Liberec, Czech Republic; jakub.erben@tul.cz (J.E.); jiri.chvojka@tul.cz (J.C.)

**Keywords:** nanofibers, neonicotinoids, online SPE, Lab-In-Syringe, membrane preconcentration

## Abstract

Polymeric nano- and microfibers were tested as potential sorbents for the extraction of five neonicotinoids from natural waters. Nanofibrous mats were prepared from polycaprolactone, polyvinylidene fluoride, polystyrene, polyamide 6, polyacrylonitrile, and polyimide, as well as microfibers of polyethylene, a polycaprolactone nano- and microfiber conjugate, and polycaprolactone microfibers combined with polyvinylidene fluoride nanofibers. Polyimide nanofibers were selected as the most suitable sorbent for these analytes and the matrix. A Lab-In-Syringe system enabled automated preconcentration via online SPE of large sample volumes at low pressure with analyte separation by HPLC. Several mat layers were housed in a solvent filter holder integrated into the injection loop of an HPLC system. After loading 2 mL sample on the sorbent, the mobile phase eluted the retained analytes onto the chromatographic column. Extraction efficiencies of 68.8–83.4% were achieved. Large preconcentration factors ranging from 70 to 82 allowed reaching LOD and LOQ values of 0.4 to 1.7 and 1.2 to 5.5 µg·L^−1^, respectively. Analyte recoveries from spiked river waters ranged from 53.8% to 113.3% at the 5 µg·L^−1^ level and from 62.8% to 119.8% at the 20 µg·L^−1^ level. The developed methodology proved suitable for the determination of thiamethoxam, clothianidin, imidacloprid, and thiacloprid, whereas matrix peak overlapping inhibited quantification of acetamiprid.

## 1. Introduction

Neonicotinoid insecticides (NNIs) are neuro-active insecticides chemically related to nicotine. They are widely used to protect mainly agricultural plants and livestock from pest insect attacks. They act on the nicotinic acetylcholine receptors in the central nervous system of the insects leading to their paralysis and ultimately to death [1].

NNIs are used worldwide due to their high efficiency and low risk to mammals and are replacing former insecticides such as organophosphates, carbamates, and pyrethroids [2]. In 2015, NNIs were registered in 120 countries worldwide and represented 25% of all pesticides used, making them the number one group of insecticides [3]. In addition, they are versatile in application and can be used as foliar spraying, seed dressing, seed pilling, soil treatment, mixing with irrigation water in drip and drench systems, and as systemic pesticides [4]. Their high water solubility enables their taking up by the roots and leaves, and the ability to be translocated to all parts of the plant. Thus, they also potentially come into contact with non-target organisms such as birds and aquatic and terrestrial invertebrates. They also affect pollinators such as honey bees, posing a serious risk to them [5]. NNIs were related to the colony collapse disorder [6] based on the loss of bees’ navigation ability upon collecting pollen and nectar. In addition, only 5% of NNIs applied to seeds actually enters the crops, whereas around 94% penetrates the soil and surface waters, or leaches into groundwaters [7].

Considering these risks, clothianidin, imidacloprid, and thiamethoxam have been banned in plant protection products and treated seeds since 2013 [8]. The EU Commission implemented regulations restricting their application to greenhouse use in May 2018 after updated risk assessments by the European Food Safety Authority for these three NNIs [9].

To further investigate their potential environmental impact, worldwide integrated assessment has considered it essential to propose efficient analytical procedures for their monitoring in environmental samples such as river waters, where their concentration ranges at ppt-ppb levels [10]. NNIs feature specific physicochemical properties such as high water solubility, thermolability, and low volatility. Therefore, liquid chromatography combined with UV [11,12,13] and mass spectrometry (MS) detection [14,15,16] or gas chromatography with MS detection [17] are the most frequently applied techniques for the determination of NNI residues in various matrices mainly including food and environmental samples. Clean-up and preconcentration steps are usually required for sample pretreatment to improve both sensitivity and selectivity. Although various methodologies have been proposed including dispersive liquid–liquid microextraction (DLLME) [18,19], liquid–liquid extraction (LLE) [20], “Quick, Easy, Cheap, Effective, Rugged, Safe” sample preparation technique, known as QuEChERS [21], and disposable pipette tip extraction [22], off-line solid-phase extraction (SPE) remains the most common sample treatment, and various types of formats have been used apart from the classical cartridges, such as SPME [17], molecularly imprinted polymer (MIP)-SPE [23], or microextraction by packed sorbent (MEPS) [24]. The scale of sorbents applied for NNI extraction include Strata-X [25], Oasis^®^HLB [26], Extrelut-NT20 [27], and diatomaceous earth (Isolute^®^ HM-N) [28] have been studied and ion-pairing is often used to achieve high extraction efficiency.

To the best of our knowledge, online SPE of NNIs has been reported only once, by Montiel-León et al., employing an automated HyperSep Retain PEP coupled to UHPLC-MS/MS [29]. Effective and complete transfer of all analytes retained on the sorbent to the separation process and their quantification is the main advantage of online SPE, while simple and automated workflow at low costs due to sorbent reusability is noteworthy.

The focus of current analytical research concerns novel sorbents and aims at improvements in selectivity, extraction effectivity, customized modifications, easy handling, speed of extraction, and sensitivity, e.g., by an increased sample-to-extractant ratio. In this context, nanofibers are gaining attention from the analyst community. Their generous surface-to-volume ratio and large interstitial voids together with options for the chemical modification to target a specific analyte make them a promising candidate to fulfil all the above-mentioned demands. Apart from that, nanofibers have proven their advantages over the classical sorbents in terms of reusability, selectivity, and feasible integration in the workflow even in online systems for easy-to-carry-out methods [30,31,32].

Our present work aimed to develop a methodology for NNI determination using HPLC with the sample preparation comprising online SPE using electrospun polymer nanofibers in mat format. In contrast to former works [30,31,32], we aimed for a low-pressure system to enable loading of large sample volumes and consequently, the nanofibrous sorbent was used in a membrane format that has been proven effective in the screening of extraction efficiency of polymeric nanofibrous sorbents for 17 analytes [33]. In the present work, the concept of the automation technique, Lab-In-Syringe (LIS) [34,35], was employed for pre-load modification of milliliter volumes of sample as well as for handling the washing and conditioning solutions. Such hyphenation of automated and simple sample preparation steps with the separation techniques enabled minimization of manual sample handling and related errors, thus improving both time management and precision. LIS represents a versatile alternative to robotic autosamplers and is easier to set-up and configure. The void of an automatic syringe pump as a closed, sealed, and size-adaptable vessel represents a significant advantage. Placing a magnetic stirring bar inside enables in-syringe, on-demand, homogenous, and nearly instantaneous mixing of the liquid content. Therefore, this technique is ideally suited for the automation of standard operation procedures such as preparation of solution, solvent extractions, solution measuring, transfer, and sorbent loading all at low to moderate pressure as required for the task set.

The nanofibrous sorbent was used in this work in a disc format cut from mats that permitted fast loading of a milliliter volume of sample, high preconcentration factor, and analyte stacking. Use of nanofibers in mat format was previously investigated by our group [36]. Yet, this is the first report on its use in online SPE with HPLC for the determination of NNIs in surface waters.

## 2. Materials and Methods

### 2.1. Reagents and Samples

Ultrapure water generated by a Millipore purifying system (18.2 MΩ cm, Millipore Corporation, Bedford, MA, USA) was used throughout the experiments. Methanol (MeOH) and acetonitrile (ACN), both LC-MS grade, as well as formic acid (p.a.) and acetic acid (p.a.), were obtained from VWR International s.r.o. (Stříbrná Skalice, Czech Republic). Analytical NNI standards acetamiprid (ACP), clothianidin (CLT), imidacloprid (IMI), thiacloprid (TCP), and thiamethoxam (TMX) were purchased from Merck (Darmstadt, Germany). Their structures and physicochemical properties are summarized in Figure S1 (Supplementary Material, SM).

Individual stock solutions of the five NNIs were prepared in MeOH at a concentration of 500 ppm. An intermediate mixed standard in water was prepared from these standards by appropriate dilution to a final concentration of 10 ppm each. This solution was daily further diluted with water to obtain standard working solutions. All solutions were kept in the dark at 4 °C when not used.

The buffer solutions of the following components and pH values were prepared at a concentration 0.1 mol L^−1^ to determine the loading conditions on the nanofibrous sorbent: formic acid (pH 2.0, 3.0, and 4.0), acetic acid (pH 5.0 and 6.0), and tris(hydroxymethyl)aminomethane hydrochloride (TRIS-HCl) (pH 7.0, 8.0, 9.0, 10.0) that were adjusted with 0.3 mol L^−1^ NaOH. In addition, 0.1 mol L^−1^ HCl was tested for sample acidification.

Gradient in HPLC elution was formed by the mobile phases, A 10% ACN (*v/v*) in 0.05% (*v/v*) aqueous formic acid and B 70% ACN (*v/v*) in 0.05% (*v/v*) formic acid. They were filtered through a 0.45 µm hydrophilic PTFE filter (Millipore Corporation, Bedford, MA, USA).

Three dimensional printed auxiliary materials were produced by fused deposition modelling (FDM) using a DeltiQ, size M printer from TriLAB Group s.r.o. (Hradec Králové, Czech Republic) and polypropylene and polylactic acid filaments.

Surface waters were collected in glass bottles in the surroundings of Hradec Králové, Czech Republic in August 2020, from one lake and three rivers, two of those from intensively agriculturally used areas. The samples were filtered through a standard filter paper, stored in the dark at 4 °C, and then used without further modification.

### 2.2. Preparation of Nanofibers

Fibrous sorbents included nanofibers from polycaprolactone nanofibers (nPCL), polyvinylidene fluoride (nPVDF), polystyrene (nPS), polyamide 6 (nPA6), polyacrylonitrile (nPAN), and polyimide (nPID) nanofibers, polyethylene microfibers (µPE) and combinations of micro- and nanofibers including (µ/nPCL), and polycaprolactone microfibers in combination with polyvinylidene fluoride nanofibers (µPCL/nPVDF)). Production of these fibers except nPID is detailed elsewhere [37,38,39]. Briefly, nPVDF, nPA6, nPS, nPAN, and nPID nanofibers were produced by electrospinning from a polymer solution in a suitable organic solvent or solvent mixture at a final concentration ranging from 7 to 16 w/t %. Taylor cone formation and fiber splintering by electrostatic repulsion yields fibers of characteristic diameters around 200 nm. µPE fibers were produced by melt-blown technology that was based on extrusion of the melted polymer through a spinning head with gaps, each 0.4 mm in diameter, resulting in the formation of microfibers (a few µm in diameter) in a hot airstream [38]. Fibers µ/nPCL and µPCL/nPVDF were produced via a novel combination of the electrospinning and melt-blown technology [37] so that the higher structural rigidity of the microfibers and the large surface due to nanosized fibers was possible to combine.

The nPID fibers (chemical structure shown in Figure S1) eventually chosen for this work were prepared via electrospinning from a 16% (*w/w*) solution of polyimide pellets P84TM SG (HPpolymer Inc., Lenzing, Austria) in N,N-dimethylacetamide (99.8%; PENTA Chemicals, Prague, Czech Republic) under conditions detailed in Table S2. In short, the solution was stirred for 24 h at 250 rpm and 22 °C and then filled in a 15 mL cartridge attached to the spinning electrode. A Nanospider extruder, type NS 1WS500U (Elmarco Ltd., Liberec, Czech Republic), was used for the electrospinning process. An air conditioning system, NS AC150 1000/2000 (Elmarco Ltd., Liberec, Czech Republic), was used to keep the desired temperature and humidity. Morphological characteristics were evaluated as detailed in Figure S2, with results shown in Figures S3–S5 and summarized in Table S3. The fiber mat was, on average, 60 µm thick with fiber diameters of about 234 nm yielding average pore diameters in the fiber mat of 0.54 µm. The porosity of the mat was evaluated to be 82.7% with an active surface area of nearly 17 m^2^ per gram sorbent.

### 2.3. Instrumentation

The LIS system used for liquid handling was assembled from an automatic Cavro XC3+ syringe pump (Tecan Trading AG, Männedorf, Switzerland) equipped with a 2.5 mL glass syringe and a 3-way head valve, an 8-port selection valve for the selection of solutions, and a 6-port high-pressure injection valve that acted as an interface between the LIS system and the HPLC system used for analyte separation and quantitation. Both external valves (SV, drive EMMA, head 4468 and IV, drive ETMA, head C2-2346D) were purchased from Vici Valco Instruments Co Inc. (Schenkon, Switzerland).

**Figure 1:**
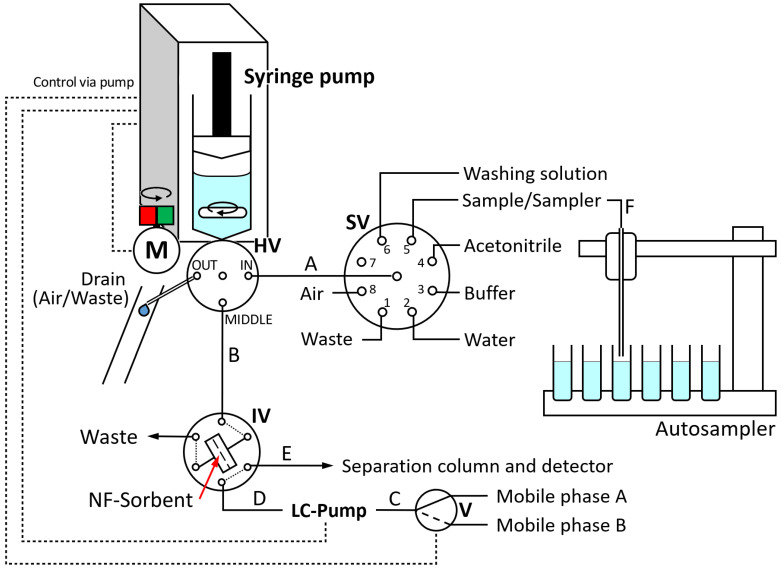
Scheme of Lab-In-Syringe system for large-volume SPE on nanofibrous (NF) sorbent membranes. HV—Head valve of syringe pump, IV—Injection valve, M—Motor, SV—Selection valve, V—Solenoid valve. Tubes: A—PTFE, 25 cm, 0.8 mm i.d., B—PTFE, 40 cm, 0.5 mm i.d., C—PTFE, 15 cm, 1.5 mm internal diameter (i.d.), D—PEEK, 40 cm, 0.2 mm i.d., E—PEEK, 33 cm, 0.2 mm i.d.

An AIM 3200 autosampler from AIM Lab Inc. (Virginia, QLD, Australia) was connected to the flow system during sample measurements to allow the automatic exchange of sample solutions. All low-pressure connections consisted of 0.8 mm i.d. PTFE tubing, whereas the high-pressure connections were PEEK capillary. Figure 1 shows the entire instrumental setup including tubing dimensions.

A magnetic stirring bar (10 mm long, 3 mm in diameter) was placed inside the void of the syringe pump to enable in-syringe homogenous mixing of the sample and loading buffer and for in-system preparation of washing solutions. A DC motor adapted from a pulse-width modulated computer fan was positioned close to the syringe [40]. The motor held a stack of neodymium magnets (25 mm, 4 mm in diameter) on top. Upon initiating the motor, the magnetic stirring bar inside the syringe followed the rotating magnetic field, thus forcing a synchronized rotation. Velocity was controlled via a simple analogue control board.

A stainless-steel PREP column in-line filter (AF0-7866, Phenomenex Int., Torrance, CA, USA) equipped with a 2 µm porosity stainless-steel filter disk (3 mm × 21.2 mm in diameter) was used for holding several layers of nanofibrous sorbent mat that were cut with preparation scissors to fit the size of the frit.

Using several layers of nanofibers, we observed mobile phase leakage since the rubber rings of the holder did not exert sufficient pressure on the fibers to seal the assembly tightly. It was also observed that the porous steel disc in combination with the fibers caused significant backpressure that prevented fiber loading via the syringe pump at a reasonable flow rate > 1 mL/min. Therefore, a disc made of a commercial felt pad purchased from a local hardware store (3 mm thickness, 22 mm in diameter), was used instead. The glue from the adhesive side was removed by soaking the felt disk repeatedly in ethyl acetate under sonication.

**Figure 2:**
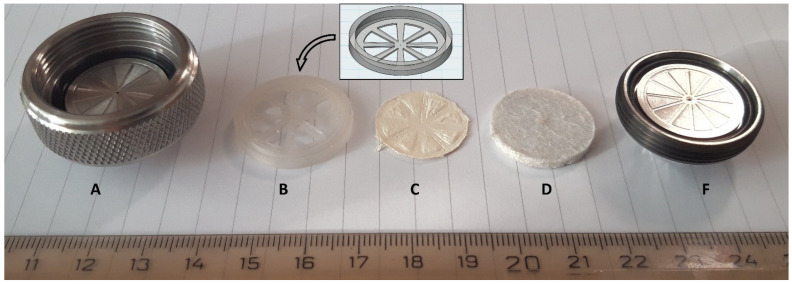
Assembly of the fiber holder consisting of a commercial in-line filter (**A**–**F**), nanofibrous sorbent (**C**), a fused deposition modelling 3D-printed holder ((**B**), design shown in box) allowing the insertion of the nanofiber mats and a commercial felt pad as support of low flow resistance.

The felt pad was inserted into a 3D printed support ring of polypropylene before placing it in the holder to obtain a hard rim that would allow high pressure sealing against the rubber rings of the in-line filter holder (Figure 2). This implementation also allowed the adaptation of the felt pad diameter to the holder dimensions. The in-line filter, in the following referred to as “fiber holder”, was integrated into the injection loop (Figure 1).

An LC-20AD pump and SPD-20A UV detector from Shimadzu Inc. (Tokyo, Japan) were used for online coupling of nanofibrous SPE automated via the LIS technique. The detection wavelength was 270 nm corresponding to the maximum absorbance of IMI and CLT. All separations were carried out using a reversed-phase fused-core Kinetex^®^ column (RP-C18 150 × 4.6, 2.1 µm, 100 Å, Phenomenex, Aschaffenburg, Germany). Gradient mode was enabled by adding a 3-way solenoid valve type MTV-3-1 UKGH from Takasago Electronics Inc. (Nagoya, Japan) on the aspiration side of the LC pump that switched proportionally between the reservoirs of mobile phases A and B to form the optimized gradient shown in Table S4. The solenoid valve was controlled via a Trinket M0 circuit microcontroller board (Adafruit, New York, NY, USA) loaded with a program written in CircuitPython programming language as reported earlier [40].

The syringe pump featured three TTL contacts that were used for relay activation/deactivation of the stirring motor, HPLC triggering, and initialization of the Trinket M0 chip for gradient operation of the HPLC.

LabSolutions software (Shimadzu Inc., Tokyo, Japan) was used for data evaluation and control of the chromatographic system. CocoSoft 5.11 [41] took care of the procedures on the flow system, i.e., sample mixing with buffer, loading, fiber washing as well as initial conditioning, cleaning of tubes, valve switching, and triggering of the chromatographic method and initiation of the gradient by the pre-programmed switching protocol of the solenoid pump.

### 2.4. Operation

The details of the operation are described in Tables S5 and S6. In short, all operation consisted of the aspiration of the required solutions from the selection valve in the syringe and propelling them slowly through the head valve in position “MIDDLE” towards the nanofibrous sorbent with the injection valve in position “LOAD” or in case of cleaning, rapidly through the head valve position “OUT” to waste.

The nanofibrous sorbent was cleaned with 1 mL ACN and 1 mL water before aspiration of 2 mL sample and 0.3 mL buffer in the syringe void with activated stirring to achieve homogeneous mixing and then loaded on the nanofibers. Afterwards, the syringe was cleaned twice with water to wash away any remnants of the sample. Then, the fibers were washed with 1 mL in-syringe diluted loading buffer. Finally, the injection valve was switched to position “INJECT” and the gradient, HPLC pump operation, and data acquisition were triggered.

Next, the analytes were eluted from the fibers in the separation column using the mobile phase with steadily increasing elution strength and their separation occurred within 14 min. The injection valve was switched 300 s after the injection back to position “LOAD” and the preconcentration of the next sample was carried out in the LIS system in parallel to the running separation.

### 2.5. Instrumentation and Methodology Used for PID Fiber Characterization

Morphological characterization: The structure of nanofiber sorbents was assessed from images obtained with an Ultra Plus (Zeiss, Germany) scanning electron microscope (SEM) using an integrated in-lens secondary electron detector at an acceleration voltage of 2 kV, an aperture of 20 µm, a working distance of 2.7 mm, and a pixel size of 44.66 nm. The dry samples were coated with a 5 nm-thick gold layer prior to the analysis.

The morphology of the materials was evaluated using NIS Elements (Nikon, Tokyo, Japan) image analysis software. Five SEM images at a magnification of 10.000, taken at different locations on the respective sample were used for the measurement of the fiber diameters. A total of 500 fibers were measured for each sample and data subjected to detailed statistical analysis.

The pore size distribution of the sorbents was determined by means of the bubble point test using a 3G zH (Anton Paar, Graz, Austria) capillary flow porometer. Three disk-shaped samples of 47 mm in diameter and measured areas of 500 mm^2^ were tested at a pressure range of 0 to 1 MPa in mineral oil.

The gravimetric method was used for the determination of the total porosity, surface density, and layer thickness. The thickness of 100 × 100 mm sample cuts was measured using a 49–63 bench micrometer (TMI, Reston, VA, USA) with a pressure of 400 Pa according to the standard EDANA testing method—NWSP 120.1.R0 (15). The weight and volume of the measured samples set in relation to the known density of the nPID (1.42 g cm^−3^) allowed estimation of both the other parameters.

The morphology of the nPID fibers was examined by scanning electron microscopy. Examples of the resulting images are shown in Figure S2. The structure was highly homogeneous with long and continuous fibers. The average fiber diameter, average pore diameter, and total porosity data for the electrospun PID nanofiber sorbent are summarized in Table S3.

Surface characterization: The sessile drop technique was used to determine the contact angle of the materials using deionized water as the wetting medium. The contact angle was measured by means of a See System 6.2 goniometer (Advex Instruments, Brno, Czech Republic). A micrometer pipette was used to deposit a 6 µL water drop onto the surface of the nanofibrous material. A couple-charged device camera was utilized to capture the behavior of the drop on the planar mat. The tangent angle at the three-phase contact point on the sessile drop profile was directly measured from the optical image. All experiments were carried out at ambient temperature (21 ± 2 °C). The measurement process was repeated twenty times from which an average value and standard deviation were calculated.

The specific surface area was determined by gas adsorption isotherm method via the Brunauer–Emmett–Teller equation using an Autosorb iQ (Quantachrome, Haan, Germany) device in standard mode. Fibrous samples (1000 mg) were placed in 12 mm glass cells and degassed for 24 h at 50 °C prior to measurement. Krypton was used for the analysis and the data was processed using ASiQwin software.

Thermic properties: The thermic stability of the PID was tested via thermogravimetric analysis (TGA) on a Q500 thermogravimetric analyser (TA Instruments, New Castle, DE, USA). The experiment was carried out in a synthetic oxygen atmosphere at a flowrate of 60 mL min^−1^ in a temperature range from 25 °C to 750 °C and a heating rate of 10 °C min^−1^.

The glass transition temperatures of the material were evaluated by differential scanning calorimetry (DSC). A 1/700 calorimeter (Mettler Toledo, Greifensee, Switzerland) was used for the measurements that were performed using 10 ± 0.5 mg of fiber samples under an N_2_ atmosphere with a heating rate of 10 °C min^−1^ from −50 °C to 350 °C. The analysis was carried out under an N_2_ flow rate of 50 mL min^−1^. The transition temperatures were taken from the second heating run.

## 3. Results

### 3.1. HPLC Conditions

The HPLC separation was optimized in off-line mode using aqueous standards. The five analytes were eluted in the order TMX, CLT, IMI, ACP, and TCP. Using MeOH as organic solvent the peak resolution of CLT and IMI was always unsatisfactory, whereas using ACN allowed baseline separation of all compounds in 8 min using the optimized gradient conditions at a flow rate of 1 mL min^−1^. The flow rate was reduced for online SPE-HPLC, i.e., system with the added inline filter, to 0.8 mL min^−1^ to counteract the increased flow resistance.

Addition of 0.05% (*v/v*) formic acid to the mobile phase improved the peak shape, resolution, and column efficiency compared with a no-acid-containing mobile phase counterpart. Doubling the formic acid concentration did not bring any further improvement. Addition of 5 mmol L^−1^ ammonium acetate was also tested but no improvement in peak resolution and average peak symmetry values were observed. Thus, addition of 0.05% (*v/v*) formic acid in the mobile phase was adopted in the final procedure.

### 3.2. Mat Holder

In contrast to former works using fiber-filled cartridges, i.e., fibers in transversal direction, the possibility to use nanofibers as a sorbent membrane was studied. An anticipated benefit was the automation of fiber loading via a flow system that would provide a larger volume than typical in HPLC integrated autosamplers. Another expected advantage was on-system sample modification while allowing operating at low pressure. Moreover, the possibility to use a smaller sorbent quantity by providing better accessibility of the fiber mat surface and consequently analyte stacking within a narrow zone were regarded. We aimed for a membrane holder with a wide diameter to accommodate a sufficient quantity of fibrous sorbent without significantly increasing the backpressure.

The in-line filter, selected to accommodate the nanofibers, was chosen for its large cross-sectional area of approximately 350 mm^2^. The original stainless-steel frit only allowed co-direction operation of loading and elution due to increased overpressure as the nanofiber mats covered the fine pores (2 µm). The use of a commercial felt pad (see Section 2.3) as a frit of higher porosity and low flow resistance combined with the polypropylene cover allowed firm holding of the nanofiber layers. This implementation enabled both adequate sealing without using the additional filter paper, as well as counter-direction of loading (nanofibers→frit) and elution (frit→nanofibers) by the mobile phase. This mode proved superior in terms of peak symmetry and area due to a significantly reduced dead volume in the elution step.

### 3.3. Selection of Nanofibers

The key parameter of method development was finding a suitable nanofibrous sorbent. Nine fibrous materials listed in Section 2.2 were examined using the LIS system for automated and reproducible loading and elution in co-direction (use of stainless-steel frit) for off-line analysis by HPLC. We opted for offline measurement in this early stage of method development to prevent any bias by uncomplete analyte elution due to unoptimized conditions.

The experiment was carried out using three layers of the respective fiber mat. The sorbent layers were placed between two layers of laboratory filter paper for easy handling, avoiding layer folding by electrostatic charge, for mechanical protection, and for improved sealing. The effect of two filter paper sheets was evaluated as blank. The fibers were washed inside the holder by the LIS system with 1 mL ACN and then 1 mL water for cleaning and conditioning. The NNI metabolite, 6-CNA (pKa = 3.73), was primarily included in our experiments, which was another reason to aim at an acidic loading pH. After loading, the retained analytes were eluted with 1 mL ACN that was previously aspirated in the syringe and then pushed through the fiber holder. In this step, the eluate was manually collected and analyzed off-line using HPLC with an injection volume of 25 µL.

**Figure 3:**
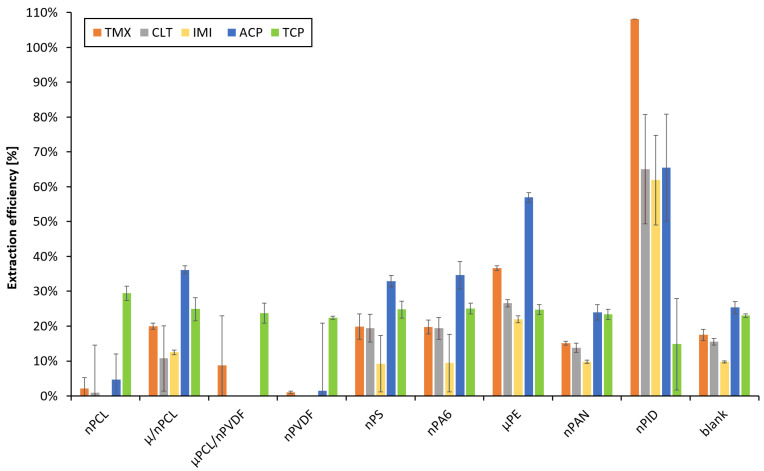
Suitability of nanofibrous sorbents for NNI compounds. Loading: 2 mL mixed standards, 50 µg·L^−1^ each, acidified with 200 µL HCl, pH 3. Elution: 1 mL ACN. Loading and elution flow rates: 500 µL min^−1^. Off-line HPLC measurement, injection volume: 25 µL.

Experiments were conducted in triplicate and the peak areas were compared with those obtained via direct injections of 100 µg·L^−1^ mixed standard to calculate the extraction efficiencies. The results presented in Figure 3 demonstrate that the highest extraction efficiencies were achieved with PID nanofibers. Extraction efficiencies between 20% and 60% were achieved with µPE. In contrast, nPS, nPA6, nPAN, and µPCL/nPVDF were not suitable for extraction of NNIs at all, as the extraction efficiencies were less than 30%. Even worse results were achieved with nPCL and nPVDF nanofibers in which only less than 10% of the target analytes were retained. The filter paper itself exhibited no significant extraction capacity for the NNIs. We further verified that the felt material used in the following experiments also did not display any significant extraction capacity for the target analytes. The slightly hydrophilic nPID (Figures S2 and S4) was selected as the extracting material for further experiments.

### 3.4. Online SPE Conditions

Sample volume was examined in offline mode after selecting the extraction sorbent. The preconcentration system was coupled to HPLC and the experiments concerning the SPE conditions were conducted online.

#### 3.4.1. Sample Volume

In a preliminary experiment, the extraction capacity of the fibers was determined using a mixed standard featuring a high analyte concentration of 1 ppm per analyte. The syringe size used in this experiment was 2.5 mL. Thus, repeated execution of the loading step was carried out when volumes exceeding 2 mL were needed before elution in online mode. The results confirmed that the response for most analytes increased linearly with the loaded volume of standard over a range of at least up to 6 mL. The extraction efficiencies remained stable at around 80% for TCP, ACP, IMI, and CLT. However, they decreased for the most polar compound TMX. Details are presented in Figure S6. These experiments verified that the sensitivity can be easily improved by simply increasing the sample volume and that the extraction capacity of the sorbent is sufficient even for absolute analyte amounts of at least 30 µg. The sample volume selected for this study was 2 mL.

#### 3.4.2. Flow Rate of Sample Loading

**Figure 4:**
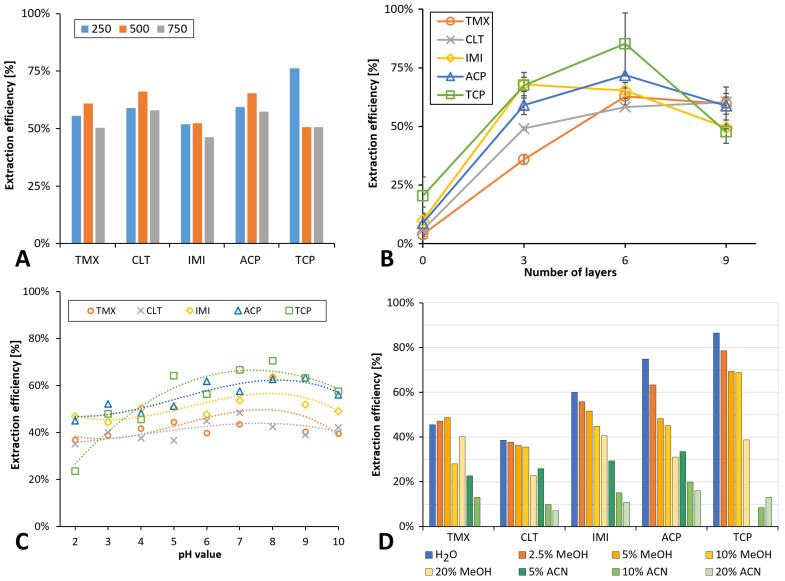
Effect of conditions on online SPE. (**A**) Flow rate at sample loading: 3 layers of PID nanofibers, loading 2 mL mixed standard, 50 µg·L^−1^, pH adjusted to 3, washing with 2 mL water; n = 2. (**B**) Number of PID nanofibers layers: loading 2 mL mixed standard, 50 µg·L^−1^, acidified with HCl 50 mmol L^−1^, washing with 2 mL water. (**C**) Loading pH value: loading 2 mL mixed standard, 10 µg·L^−1^, with in-syringe addition of 300 µL buffer (pH 2–4—formate, pH 5 and 6—acetate, pH 7–10—TRIS-HCl). Washing with 2 mL water mixed in-syringe with 50 µL buffer. (**D**) Composition of washing solution: 6 layers PID nanofibers, loading 2 mL mixed standard, 10 µg·L^−1^, acidified with HCl 50 mmol L^−1^.

The flow rate for sample loading was studied at 250, 500, and 750 µL min^−1^. Figure 4A shows the parameters and results. We found that the slowest flow rate, providing the longest contact time of the analyte with the nanofibers, did not provide any benefit compared with a flow rate of 500 µL min^−1^ for four out of five analytes but even the reproducibility decreased. On the other hand, a decrease in the extraction efficiency was observed after an increase in the flow rate. Therefore, an intermediate value of 500 µL min^−1^ was chosen for further work that led to both acceptable extraction efficiency and time of analysis.

#### 3.4.3. Number of PID Layers

An increase in the extraction efficiency was the main objective of all following experiments. The amount of the sorbent, or rather the number of nanofiber layers, was considered to have a substantial effect on the method sensitivity and extraction efficiency particularly in terms of the extraction capacity considering the short contact time of the analytes with the sorbent. Results achieved with stacks of three, six, and nine PID layers were compared with an extraction carried out with the felt pad alone, i.e., without any PID layer (Figure 4B). The extraction recovery increased up to six layers, achieving values between 58% (CLT) and 85% (TCP), whereas nine layers yielded only similar results to those using a mere three layers. We assume that the reason for this phenomenon is a reduced penetration of both sample and eluent through the fibrous layers that decreases the accessibility of the fiber surface as well as delayed elution of the analytes from the larger mass of fibers. Therefore, six layers were used in further experiments.

#### 3.4.4. Loading pH and Salt Addition

Previous works reported NNI extraction in acidic medium yet using SDS as ion-pairing reagent [42]. Initially, we opted for an acidic loading pH with the aim to co-extract NNI metabolite 6-chloronicotinoic acid (6-CNA), and also considering the fact that at least TMX, CLT, and TCP are neutral between pH 2 and 9 (Table S1). However, finding an insufficient extraction efficiency in the PID fibers for 6-CNA, we decided to study the effect of the loading pH over a range of pH 2–10 using 300 µL buffer solution mixed inside the syringe with 2 mL sample. Figure 4C shows that the extraction efficiency increased slightly with an increase in the loading pH to pH 8 and then decreased for higher values. We decided to use TRIS buffer pH 8, which yielded the highest extraction efficiency, for all further work.

In addition, we examined reducing the water solubility of the analytes and increasing their affinity to the fibrous sorbent via increasing the ionic strength of the loaded sample solution. For this, standards were prepared in water as well as in 30% (*w/w*) NaCl solution. No significant salting-out effect was observed (data not shown). Thus, this strategy to increase the extraction efficiency of the NNI analytes was not adopted.

#### 3.4.5. Washing Solution

The effect of the type and quantity of the washing solution used after sample loading on the analyte recovery was assessed. Diluted MeOH and ACN, 2 mL, were considered as washing solutions at concentrations of 5, 10, and 20% (*v/v*), and were compared with neat water (Figure 4D). An MeOH content of 2.5% (*v/v*) did not cause any significant loss of analytes. At 5% (*v/v*) MeOH, a signal decrease of about 20% was observed only for TCP. ACN eluted part of the analytes even at the lowest tested concentration. Therefore, a washing solution of 2.5% (*v/v*) was used for further experiments.

The volume of the washing solution had to be large enough to eliminate the remains of sample from the injection loop including the dead volume of the sorbent holder, yet, as small as possible to minimize waste, time of analysis, and to avoid untimely elution of retained analytes. For the related experiment, a two-times-higher concentration of the standard (20 µg·L^−1^ of each analyte) was used to facilitate verifying that the washing solutions sufficed to wash out any residues of the non-retained analytes. In addition, the washing solution was adjusted to the optimal loading pH by adding 2.5% (*v/v*) of the loading buffer to the MeOH washing solution.

Peak areas decreased on average by 25% after increasing the washing solution volume from 750 µL to 2000 µL, accounting for 5% with each additional 250 µL. Therefore, 1 mL washing solution (2.5% *v/v* MeOH in 2.5 mmol L^−1^ buffer) was chosen for following experiments. Decreasing the volume of washing solution and using a flow rate of 750 µL min^−1^ for sorbent washing reduced the time of this step from 2.6 to 1.3 min.

### 3.5. Analytical Figures of Merit and Analysis of Surface Waters

Repeated calibrations, as well as analysis of surface water, both native and spiked with analyte standards, were measured to evaluate method characteristics including signal linearity, reproducibility, and applicability of the optimized method. Table 1 summarizes the obtained analytical figures of merit. The average extraction efficiency was 77.0 ± 4.8%. Extraction efficiencies were calculated by comparing the peak areas of 2 mL 10 µg·L^−1^ standards undergoing the online SPE-HPLC procedure and peak areas of 1 ppm standards after direct injection of 20 µL.

The values of limits of detection and quantitation were calculated from the threefold and tenfold amplitude of the baseline divided by the slope that is given in Table 1 together with the relative standard deviations of peak areas including the extraction step. LOD values were between 0.4 and 1.7 µg·L^−1^, thus meeting the requirements for method sensitivity for neonicotinoids in water [10]. Repeatability of the entire procedure was typically below 5%. Preconcentration factors were 70 to 82 corresponding to an equal percentage extraction efficiency considering a theoretical preconcentration factor of 100 (injection loop size for direct injection: 20 µL vs. online preconcentration of 2 mL sample).

The observed validated parameters of the separation method, i.e., the peak width at 5% peak height and the tailing factor, were somewhat affected by online SPE, yet to a justifiable degree. Whereas peak resolution was satisfactory throughout, with values from 2.9 to 8.3, the observed dispersion caused by the dead volume of the holder caused peak broadening by about 30% (0.212 min on average compared with 0.165 min for direct injection) and a peak symmetry/tailing factor of 1.7 on average with the extraction step compared with 1.2 for the direct injection. The nanofibers’ contribution to this increase was negligible and was mainly caused by the holder itself, whereas the felt pad did not contribute due to applying elution in counter-direction. On the other hand, the implied dead volume was considered to have affected the repeatability of retention times. These were adequate for sample analysis (average value 4.9% RSD, n = 6) yet missed SANTE/12682/2019 European legislation requirements for pesticide analysis [43]. The obtained HPLC peak parameters are listed in Table S7.

**Figure 5:**
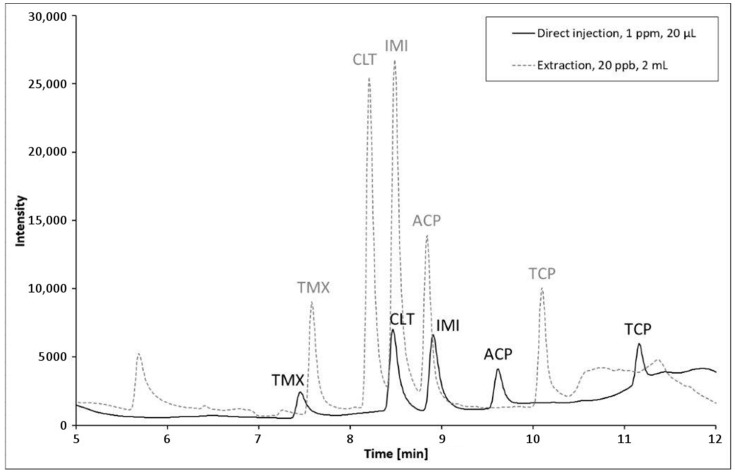
Chromatograms of direct injection and online preconcentration with nanofibrous sorbent.

The analyzed surface waters were collected in August 2020 in the area of Hradec Králové, Czech Republic. Sample 2 was collected at a former excavation lake. The other three samples were collected from rivers and ditches in the same area. The only sample treatment carried out was filtration through standard filter paper to eliminate sedimented and suspended particulate matter. Neonicotinoids were not found in any of the collected samples. To estimate the method applicability, the samples were spiked with 5 or 20 µg·L^−1^ mixed standard solutions, to mimic contaminated surface waters. The results are listed in Table 2 and chromatograms of spiked sample compared with direct injection of 1 ppm standard are given in Figure 5.

It was found that quantifying ACP was not possible due to an overlap of the compound and matrix peaks that could not be resolved. On the other hand, analyte recovery values for the other analytes calculated from the calibration curves ranged from 53.8 to 113.3% for a 5 µg·L^−1^ spike level, with the exception for CLT in Sample 3, and from 62.8 to 119.8% for samples spiked to 20 µg·L^−1^. Repeatability values were slightly higher than in the standards measurement but generally did not exceed 10% RSD. It must be said that recovery levels were not satisfactory at the 5 µg L^−1^ level, i.e., <60% in five cases, which for one suggests that the actual LOQ value for the studied matrix must be estimated higher than values predicted from baseline noise and method sensitivity as given in Table 1. It is assumed that recovery deterioration was due to humic substances that compete with the nanofiber sorbent.

Table 3 presents an overview of previously reported methods including solid-phase preconcentration of NNIs followed by HPLC with spectrophotometric detection, i.e., analytical methodologies comparable with the one we proposed. While LOD or LOQs values obtained in our work were in the same range as reported by other authors, only one publication from Kachangoon et al. [44] also reported on surface water analysis using HPLC-UV. In fact, requiring five times more sample for a manual procedure, the authors obtained a similar recovery value range and LOD/LOQ values as in the present work. Preconcentration factors were calculated from the peak areas of directly injected and online preconcentrated standards and were with 70 (TMX) to 82 (IMI), reaching higher values than in most former HPLC-UV methodologies. This relatively high factor was achieved due to the large ratio of sample volumes and effective sorbent volume and confirms the benefit of the preconcentration using nanofibrous sorbent.

Moreover, we know of no other automated sample preparation method for NNIs using HPLC-UV, whereas manual procedures for LC-MS based on MIPs or MEPS that can principally be automated have been reported [23,24]. 

Most methods for NNI analysis were developed for food matrices and rely on QuEChERS or other combinations of solid-phase and liquid–liquid extractions for double matrix cleanup and analyte preconcentration (e.g., homogenous liquid–liquid extraction for matrix removal followed by analyte enrichment via SPE) that consequently achieved recoveries in a narrower range. This is explained by the complexity of sample matrices including honey, fruits, grain, and vegetables. On the other hand, a time-consuming procedure, typically dispersive SPE, is generally required including addition of buffers, solvent, vortexing, centrifugation, collecting the supernatant, supernatant evaporation and reconstitution, and a secondary clean-up. 

For instance, Campillo et al. used a C-18 functionalized sorbent with preceding DLLME. ACN used as an eluent in the first step was further used in the DLLME step as a dispersant of chloroform, which acted as extractant [12]. Wang et al. even combined QuEChERS with three sorbents for extract clean-up followed by DLLME for further preconcentration that reached only a factor of 5 [45]. In another publication, Wang et al. [23] developed a MIP-based SPME to extract six NNIs. They reached extraction factors better than those of commercial kits, however, they were lower than the EF of our proposed method. Moreover, the extraction took 150 min. SPE alone appears insufficient for clean-up of those matrices when using HPLC-UV, and only a few authors reported on this technique. For example, Moyako et al. used montmorillonite as a green and novel sorbent in dispersive SPE [46]. Despite numerous steps carried out manually, preconcentration factors range below values we achieved in the present work [11,47,48,49]. In fact, their analyte recovery values calculated from spiking experiments as well as values of LOD/LOQ and procedural reproducibility were in the same range, often even inferior to those obtained by our proposed system and method. Given the relatively high polarity of NNIs, alternative extraction media such as ionic liquids [50], micelles [44], and anionic surfactant SDS as ion-pairing reagent [42] have been used to enhance the extraction efficiency. Possible undesirable dynamic coating of the HPLC column by such extraction media that could enhance peak tailing, as well as the need for recovery and collection of very small volumes of extractant are possible drawbacks of these former approaches that can be omitted by online SPE such as performed in this work. To the best of our knowledge, no method so far has reported use of flow automation of the sample preparation step, application of nanofibrous sorbents, and online SPE with a perpendicularly permeated sorbent mat in HPLC methods. As expected, the perpendicular fiber arrangement, i.e., using the fibers as a sorbent membrane, allowed for low-pressure automation of the loading step to reach high analyte pre-concentration factors. The peak shape deterioration against direct injection due to the dead volume of the holder could be improved by proper adaptation of the membrane holder, i.e., reducing the void volume.

Methods comprising MS detection generally achieve higher sensitivity and selectivity, so a critical comparison would hardly be reasonable. However, it should be pointed out that the LOD/LOQ values achieved in this work and others listed in Table 3 are in the same order of magnitude as for some methods using MS detection. On the other hand, Zhang et al. [54] and Iancu et al. [55] achieved up to three orders of magnitude lower LOD values using LC-MS in NNI analysis of waters yet needed about 200- and 100-times larger sample volumes, respectively, to accomplish the preconcentration by SPE. 

The low recovery values found at 5 µg L^−1^ spiking levels are not possible to compensate, as it is with MS detection, using deuterated internal standards and suggest that the effective LOQ value must be considered to be somewhat higher than the values evaluated by baseline noise or will require the use of an appropriate internal standard.

We are confident that an additional cleanup step based on homogenous liquid–liquid extraction to remove humic substances would allow further improvement of the method’s reliability. A second item to fully benefit from a very thin yet efficient sorbent as the nanofiber tissue layers have proven in this research, the dead volume implied by the sorbent holder must be minimized, implying hardware optimization. 

Here, we aimed for a proof-of-concept for using a nano-fibrous sorbent in membrane format in online SPE in HPLC. The proposed HPLC method required only volatile components for mobile-phase preparation implying compatibility of the proposed methodology with MS detection to yield higher sensitivity and analyte selectivity. 

Clearly, the performance of the present method is comparable, and in part even superior to previous reports using HPLC with spectrophotometric detection, in terms of sensitivity, reproducibility, procedural time, and analyte recovery. Successful application with surface waters confirmed that the system and method were simple and effective, using solely online SPE in microscale format. Carrying out the sample preparation procedure in a fully automatic fashion and coupled online to HPLC is an unprecedented feature among the methodologies developed for NNI analysis. Following the evaluation scheme of greenness for sample preparation procedures proposed recently [56], the method yielded a value over 0.7, failing mostly by being a laboratory, i.e., off-line and not field-employed yet automated and online coupled sample preparation approach.

Polymeric nanofibrous sorbents have proven to be efficient sorbents for a wide range of analytes [30,33,36,37] so the proposed methodology could be used for other, not particle-laden matrices and analytes with the highlighted advantages of automation and large-volume loading feasible at low pressure. Downscaling of the membrane holder, the use of novel materials for nanofiber modification, and load by HPLC-integrated autosamplers able to load larger volumes are modalities to further improve our approach.

## 4. Conclusions

Polymer nanofibers were investigated for the first time as novel sorbents for neonicotinoid pesticides with polyimide being the most suitable material. Online SPE with preconcentration of large sample volume using a Lab-In-Syringe technique was then developed and successfully applied to the determination of NNIs in surface waters. The developed method enabled automated sorbent conditioning, in-system sample mixing with loading buffer, analyte preconcentration, and finally their separation and detection via a compact analyzer system. Using the nanofibers in disc format, reproducible cartridge filling, low back pressure, and preconcentration factors exceeding 70 were achieved that enabled determination of NNIs at nanomolar concentrations with just UV spectrometry as a readily available detection technique. This sensitivity fulfilled the requirements on NNI analysis in water bodies. The time efficiency of separation in the gradient mode accomplished via instrument modification and parallel operation of preconcentration and online coupled analyte separation, as well as adequate analyte recovery and sensitivity were demonstrated. The viability of the proposed system and method was confirmed by surface water analysis. Our method was comparable or superior in terms of analytical performance including sample throughput, sensitivity, reproducibility, and recovery to those using HPLC-UV and reported elsewhere. Further improvement of the extraction efficiency via modification of the surface of the fibers and widening the application to other analyte groups is foreseen in future continuation of the work.

## Figures and Tables

**Table 1:** Analytical figures of merit.

Analyte	Sensitivity/Calibration Slope (n = 3) [mV·L·µg·^−^^1^]	Linear Range [µg·L^−1^]	LOD [µg·L^−1^]	LOQ [µg·L^−1^]	Repeatability (% RSD, n = 3, 10 µg·L^−1^ Level)	Preconcentration Factor
TMX	2.16 × 10^3^ ± 24	2.0–100.0	0.55	1.82	6.8	70
CLT	6.50 × 10^3^ ± 240	2.0–100.0	0.56	1.88	0.4	76
IMI	7.00 × 10^3^ ± 56	2.3–100.0	0.67	2.25	2.8	82
ACP	2.90 × 10^3^ ± 225	5.5–100.0	1.65	5.49	4.4	81
TCP	2.35 × 10^3^ ± 119	1.0–100.0	0.36	1.21	4.2	76

**Table 2:** Recoveries of analytes in spiked samples.

Recovery [%]	Sample 1		Sample 2		Sample 3		Sample 4	
Analyte	5 µg·L^−1^	20 µg·L^−1^	5 µg·L^−1^	20 µg·L^−1^	5 µg·L^−1^	20 µg·L^−1^	5 µg·L^−1^	20 µg·L^−1^
TMX	101.7 ± 12.8	97.3 ± 4.5	113.3 ± 0.9	95.4 ± 4.6	n.e.	83.3 ± 4.9	90.2 ± 10.0	103.6 ± 22.6
CLT	74.1 ± 6.0	91.7 ± 6.8	59.0 ± 5.7	82.6 ± 2.0	37.6 ± 12.8	65.1 ± 0.7	54.1 ± 4.8	84.8 ± 8.3
IMI	85.0 ± 1.4	91.7 ± 1.0	81.2 ± 7.2	81.5 ± 0.9	83.1 ± 5.1	80.0 ± 7.3	83.3 ± 7.3	92.9 ± 5.7
TCP	60.7 ± 14.5	76.1 ± 8.0	60.5 ± 3.0	62.8 ± 7.1	53.8 ± 15.8	66.7 ± 3.5	53.9 ± 5.8	68.5 ± 2.4

n.e.—not evaluated.

**Table 3:** Overview of methods for the determination of neonicotinoid pesticides using liquid chromatography with UV detection.

Analyte	Sample Type, Quantity	HPLC Mode and Column	Injection Volume [µL]	Extraction Method	Time [min]	EF	LOD LOQ	Recovery [%]	Ref.
ACP, CLT, IMI, TCP, TMX	Honey, 2 g	Gradient Spherisorb 0DS2 (150 mm × 4 mm, 5 µm)	20	SPE with C1, then DLLME with CHCl_3_ in the ACN extract	10	13 *	0.2–1.0 µg kg^−1^ 07–3.3 µg kg^−1^	90–104	[12]
ACP, CLT, DNT, IMI, NTP, TCP, TMX	Grain (brown rice, maize, millet, oat), 10 g	Isocratic Agilent TC-C18 (250 × 4.6 mm, 5 µm)	20	QuEChERS with clean-up with PSA, C18, and graphitized carbon black followed by DLLME with CHCl_3_ + CH_2_Cl_2_	28	5 *	2–5 µg kg^−1^ 7–18 µg kg^−1^	76–123	[45]
DNT, NTP, ACP, CLT, IMI, TMX	Tea, honey, 0.1 g	Isocratic Agilent Zorbax Eclipse Plus C18 (2.1 × 100 mm, 1.8 μm)	20	MIP-SPME	25	10–56	0.03–0.58 µg L^−1^ 0.09–1.93 µg L^−1^	85.4–116.8	[23]
ACP, CLT, IMI, TCP, TMX	Fruit juice, surface waters, 13 mL	Isocratic LiChrospher^®^100 RP-18 ec (4.6 mm × 150 mm, 5.0 µm)	20	DµSPE using montmorillonite	13	8–176	0.005–0.065 µg L^−1^ 0.008–0.263 µg L^−1^	70–138	[46]
ACP, IMI, FNC, NTP, TCP, 6-CNA	Cucumber, soil, 10 g	Isocratic Synergi Hydro RP C18 (250 × 4.6 mm, 4 µm)	50	Modified QuEChERS, clean-up of ACN extract with C18	20	1 *	6–122 μg kg^−1^ 18–366 μg kg^−1^	77–120	[11]
ACP, IMI	Tomato, 2 g	Isocratic ZORBAX Eclipse Plus C18 (250 × 4.6 mm, 5 µm)	5	QuEChERS	4	1 *	3.31–8.53 µg kg^−1^ 11–28 µg kg^−1^	83–97	[47]
ACP, CLT, DNT, IMI, NTP, TCP, TMX	Honey, 5 mL	Gradient ZORBAX Eclipse XDB-C18 (50 × 4.6 mm, 1.8 µm)	Not given	DLLME with ACN and dichlormethane; QuEChERS	7	10 *	1.5–2.5 µg kg^−1^ 2.0–2.5 µg kg^−1^	73.1–118.3	[48]
ACP, IMI	Pistachio, 5 g	Isocratic Alltima C18 (250 × 4.6 mm, 5 μm)	20	Modified QuEChERS	10	5 *	10–20 μg L^−1^ 33–60 μg L^−1^	70–114	[49]
ACP, IMI, TMX	Fruit juice and vegetables, 10 mL	Isocratic STR–ODS (II) (150 × 4.6 mm, 5 µm)	5	Effervescence-assisted DLLE using an ionic liquid	8	6.65–8.4	0.12–0.33 µg L^−1^ 0.41–1.1 µg L^−1^	66–84	[50]
ACP, CLT, IMI, TCP, TMX	Surface water, 10 mL	Isocratic Chromolith^®^ HR RP-18 ec (4.6 × 100 mm)	20	Ultrasonically modified CPE with Triton X-114	9	20–333	0.3–2 µg L^−1^ 3–6 µg L^−1^	64–120	[44]
ACP, CLT, IMI, NTP, TMX	Water and fruit juice, 10 mL	Isocratic Atlantis dC18column (150 × 4.6 mm,5 μm)	20	VSLLME-SFO ** with octanol and SDS	8	20–100	0.1–0.5 µg L^−1^ 2–3 µg L^−1^	85–105	[42]
ACP, CLT, IMI, TCP	Honey, 8 mL	Isocratic LiChrosphers 100RP-18 ec (150 × 4.6 mm, 5 µm)	20	Effervescence-assisted DLLE using ionic liquid	12	50 *	0.01 µg L^−1^ 0.03 µg L^−1^	86–100	[51]
ACP, IMI, TCP	Honey, 2 g	Isocratic WondaSil C18 (250 × 4.6 mm, 5 µm)	20	Matrix-induced sugaring-out method SULLE with ACN	15	-	21–27 μg kg^−1^ 70–90 μg kg^−1^	91–98	[52]
ACP, CLT, IMI, NTP, TCP	Commercial fruit juices, 50 mL	Isocratic Zorbax SB-Aq (150 × 4.6 mm i.d., 5 μm)	10	Ultrasound-assisted DLLME with toluene	9	34–40	0.08–0.31 µg L^−1^ 0.27–0.92 µg L^−1^	68–80	[53]
ACP, CLT, DNF, IMI, TCP, TMX	Natural waters, 2 mL	Gradient, Kinetex RP-C18 (4.6 × 150, 2.1 µm, 100 Å).	n.a.	Automated online SPE using nanofibers as sorbent membrane	16	70–82	0.36–1.65 µg L^−1^ 1.21–5.49 µg L^−1^	63–120 **	Present method

* Calculated. ** Calculated at spike level 20 µg·L^−1^. Abbreviations not explained so far: DNT—dinotefuran, EF—Enrichment factor, n.a.—not applicable due to online coupling of SPE and HPLC, NTP—nitenpyram, FNC—flonicamid, CPE—cloud point extraction, VSLLME-SFO—vortex-assisted surfactant-enhanced emulsification liquid–liquid microextraction with solidification of floating organic droplet, SULLE—sugaring-out-assisted liquid–liquid extraction.

## Supplementary Materials

The following supporting information can be downloaded at: https://www.mdpi.com/article/10.3390/membranes12070648/s1, Table S1: Chemical structure and properties of the analytes., Figure S1: Chemical structure of PID nanofibers, commercial material P84^®^, Table S2: Conditions for electrospinning fabrication of PID nanofibers, Figure S2: SEM images of the PID nanofibrous sorbent, Figure S3: Fiber and pore diameter distributions of the PID nanofibrous sorbent, Figure S4: SEM image of the contact angle measurements of PID nanofibers, Figure S5: Thermic characteristics of PID nanofibers. (a) DSC curve for the glass transition temperatures determination. (b) TGA analysis of the point of thermic stability, Table S3: Material characteristics of PID nanofibers, Table S4: Time program for gradient elution, Table S5: Routine “Syringe cleaning”, Table S6: LIS method for automated nanofibrous online SPE, Figure S6: Effect of sample volume on the analytical response, Table S7: Obtained peak characteristics (5 µg·L^−1^, n = 6).
