# Supplementary material for: Nanofibrous Online Solid-Phase Extraction Coupled with Liquid Chromatography for the Determination of Neonicotinoid Pesticides in River Waters
Source: Membranes (Basel). 2022 Jun 24;12(7):648. doi: 10.3390/membranes12070648 (PMC9319645; doi:10.3390/membranes12070648)
Supplement: Supplementary file 1 [file membranes-12-00648-s001.zip › membranes-1635093-supplementary.pdf]

membranes

Supplementary Materials

Nanofibrous Online Solid-Phase Extraction Coupled with Liquid Chromatography for the Determination of Neonicotinoid Pesticides in River Waters

Ivana H. Šrámková [1,*], Burkhard Horstkotte [1], Laura Carbonell-Rozas [2], Jakub Erben [3], Jiří Chvojka [3], Francisco J. Lara [2], Ana M. García-Campaña [2] and Dalibor Šatínský [1]

[1] Department of Analytical Chemistry, Faculty of Pharmacy in Hradec Králové, Charles University, Heyrovského 1203, 500 05 Hradec Králové, Czech Republic; horstkob@faf.cuni.cz (B.H.); satinsky@faf.cuni.cz (D.Š.)
[2] Department of Analytical Chemistry, Faculty of Sciences, University of Granada, Av. Fuente Nueva s/n, E-18071 Granada, Spain; rozas@ugr.es (L.C.-R.); frjlara@ugr.es (F.J.L.); amgarcia@ugr.es (A.M.G.-C.)
[3] Department of Nonwovens and Nanofibrous Materials, Faculty of Textile Engineering, Technical University of Liberec, Studentská 2, 461 17 Liberec, Czech Republic; jakub.erben@tul.cz (J.E.); jiri.chvojka@tul.cz (J.C.)
* Correspondence: sramkovai@faf.cuni.cz; Tel.: +420-495-067-183

Citation: Šrámková, I.H.; Horstkotte, B.; Carbonell-Rozas, L.; Erben, J.; Chvojka, J.; Lara, F.J.; García-Campaña, A.M.; Šatínský, D. Nanofibrous Online Solid-Phase Extraction Coupled with Liquid Chromatography for the Determination of Neonicotinoid Pesticides in River Waters. *Membranes* **2022**, *12*, 648. https://doi.org/10.3390/membranes12070648

Academic Editors: Guorong Xu Rasel Das and Michiaki Matsumoto

Received: 26 February 2022
Accepted: 20 June 2022
Published: 24 June 2022

Publisher's Note: MDPI stays neutral with regard to jurisdictional claims in published maps and institutional affiliations.

Copyright: © 2022 by the authors. Licensee MDPI, Basel, Switzerland. This article is an open access article distributed under the terms and conditions of the Creative Commons Attribution (CC BY) license (https://creativecommons.org/licenses/by/4.0/).

Content

Table S1. Chemical structure and properties of the analytes. 2
Figure S1. Chemical structure of PID nanofibers, commercial material P84®®. 2
Table S2. Conditions for electrospinning fabrication of PID nanofibers. ..2
Figure S2. SEM images of the PID nanofibrous sorbent. 3
Figure S3. Fiber and pore diameter distributions of the PID nanofibrous sorbent. 3
Figure S4. SEM image of the contact angle measurements of PID nanofibers. 3
Figure S5. Thermic characteristics of PID nanofibers. (a) DSC curve for the glass transition temperatures determination. (b) TGA analysis of the point of thermic stability 4
Table S3. Material characteristics of PID nanofibers. 4
Table S4. Time program for gradient elution. 4
Table S5. Routine "Syringe cleaning". 5
Table S6. LIS-method for automated nanofibrous online SPE. 5
Figure S6. Effect of sample volume on the analytical response. 6
Table S7. Obtained peak characteristics (5 μg L^{-1}, n = 6). 7

Table S1. Chemical structure and properties of the analytes.

Analyte	Abbreviation	Formula	logP	pK_a of bases*	Water solubility [mg·L^{-1}]
Thiametoxam	TMX	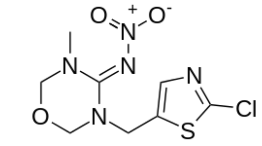	0.16	0.40	4100
Clothianidin	CLD	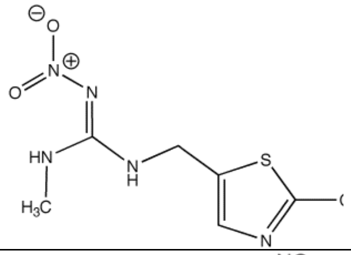	0.732	0.44<br>−0.79	340
Imidacloprid	IMI	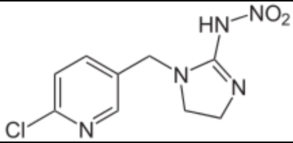	0.33	5.28<br>−0.44	610
Acetamiprid	ACP	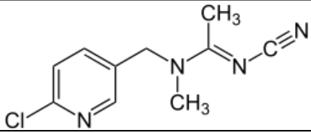	1.55	4.16<br>−0.21	2950
Thiacloprid	TCP	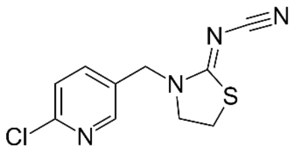	2.2	1.62<br>−0.19	184

* www.chemicalize.com.

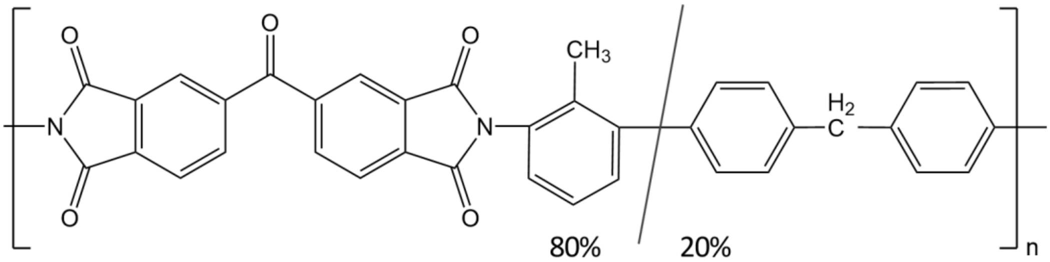

Figure S1. Chemical structure of PID nanofibers, commercial material P84®®.

Table S2. Conditions for electrospinning fabrication of PID nanofibers.

Distance electrode to collector	200 mm
Spinning electrode charge	+45 kV
Collector charge	−10 kV
Speed of polymer application	350 mm s^{-1}
Nozzle orifice size	0.7 mm
Speed of support textile movement	35 mm min^{-1}
Ambient temperature	25 °C
Humidity	10 %rel. humidity

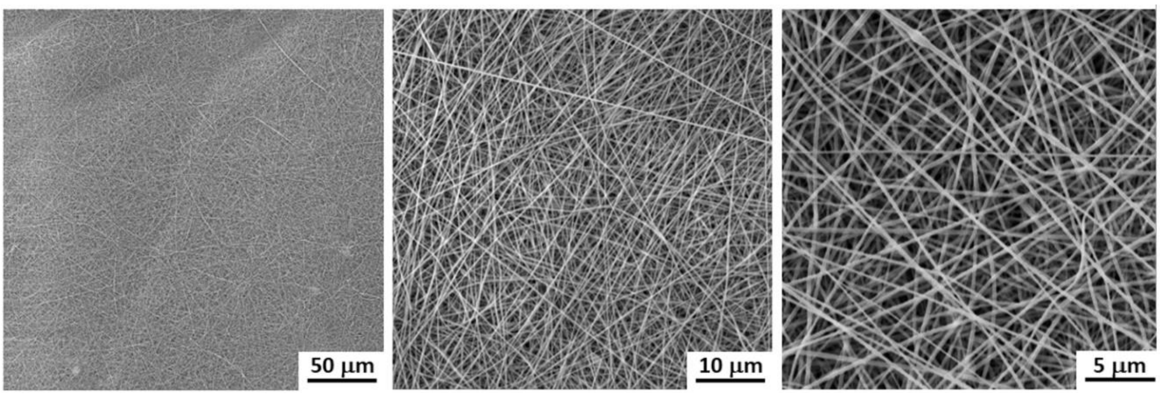

Figure S2. SEM images of the PID nanofibrous sorbent.

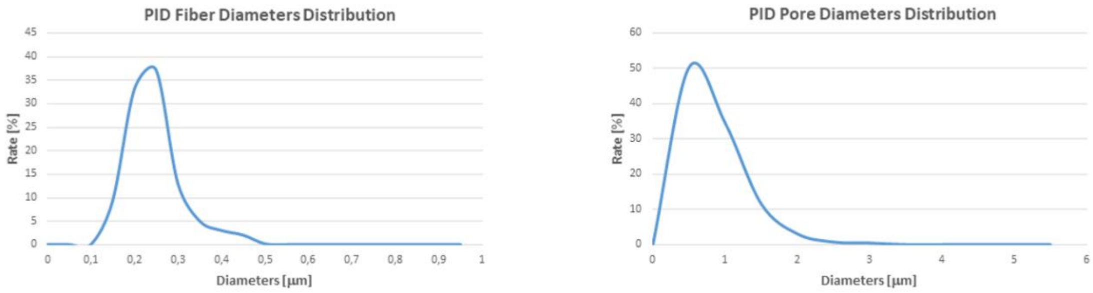

Figure S3. Fiber and pore diameter distributions of the PID nanofibrous sorbent.

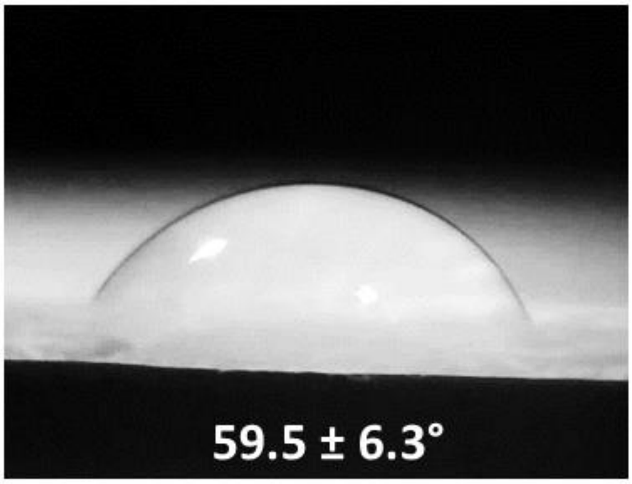

Figure S4. SEM image of the contact angle measurements of PID nanofibers.

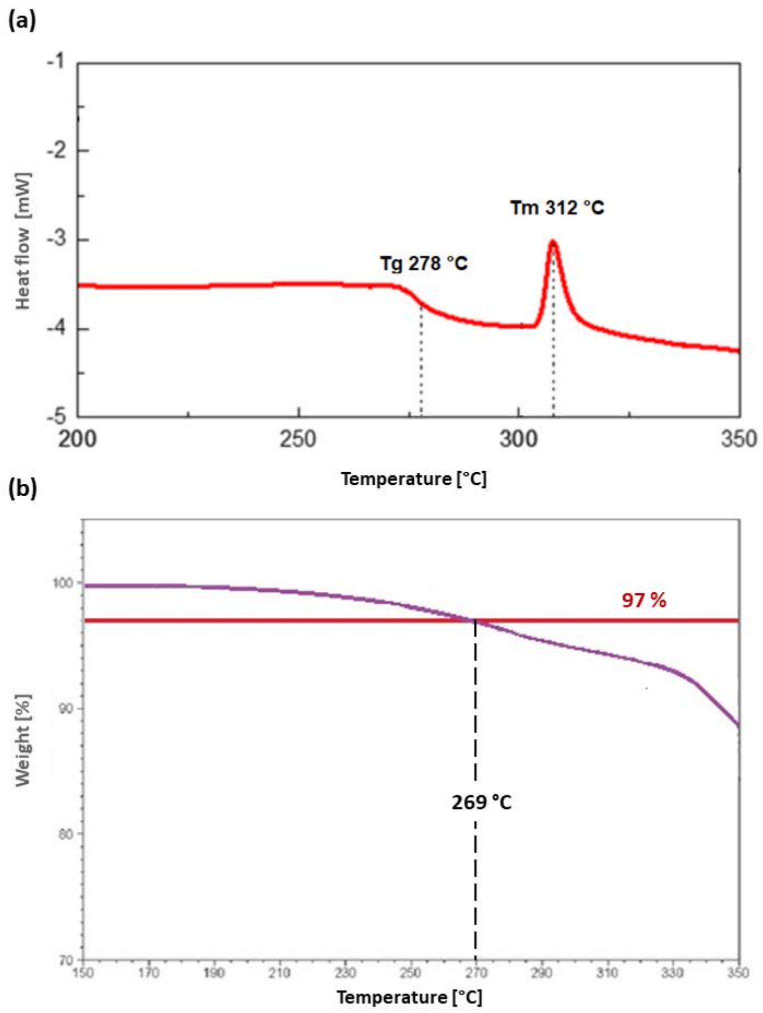

Figure S5. Thermic characteristics of PID nanofibers. (**a**) DSC curve for the glass transition temperatures determination. (**b**) TGA analysis of the point of thermic stability.

Table S3. Material characteristics of PID nanofibers.

Molecular weight of PID	60 000 g mol^{-1}
Surface density	3 g m^{-2}
Thickness of mat	60 µm
Production rate	1.93 g h^{-1}
Fiber diameters	0.234 ± 0.06 µm
Pore diameters	0.54 ± 0.09 µm
Porosity	82.69 %
Contact angle	59.50 ± 6.30 °
Surface area	16.95 m^2 g^{-1}
Thermal degradation	269°C
Glass transmission temperature	278°C
Melting point	312°C

Table S4. Time program for gradient elution.

Time [min]	0.0	1.0	6.0	6.5	7.5	8.5	11.0	14.0
Mobile phase B [%]	0	0	15	45	45	60	0	0
Content of ACN [%]	10	10	20.5	41.5	41.5	52	10	10

Table S5. Routine "Syringe cleaning".

Instruction	Comment
Selection valve to position „PORT"	Selection of respective valve port of aimed cleaning solution
Aspirate 500 µL at 10 mL/min from head valve position OUT	Aspiration of cleaning solution
Relay 1 in ON<br>Wait 5 s<br>Relay 1 in OFF	Activate for 5 s
Empty syringe at 10 mL/min to head valve position IN<br>Wait 1 s	Discharge to waste

Table S6. LIS-method for automated nanofibrous online SPE.

Instruction	Comment
Selection valve to position 4<br>Aspirate 1000 µL at 10 mL/min from head valve position OUT<br>Wait 1 s	Aspiration of acetonitrile
Injection valve to position LOAD<br>Empty at 0.75 mL/min to head valve position MIDDLE<br>Wait 3 s	Washing nanofibers with acetonitrile
Selection valve to position 2<br>Aspirate 1000 µL at 10 mL/min from head valve position OUT<br>Wait 1 s	Aspiration of water
Empty syringe at 0.75 µL/min to head valve position MIDDLE<br>Wait 3 s	Washing nanofibers with water
Selection valve to position 3<br>Aspirate 200 µL at 10 mL/min from head valve position OUT	Cleaning holding coil and syringe head valve with buffer
Relay 1 in ON<br>Wait 2 s<br>Relay 1 in OFF	Activate stirring for 2 s
Empty syringe at 10 mL/min to head valve position IN<br>Wait 1 s	Discharge buffer remains from syringe
Selection valve to position 3<br>Aspirate 300 µL at 10 mL/min from head valve position OUT<br>Wait 2 s	Buffer aspiration
Relay 1 in ON	Activate stirring
Activate stirring<br>Selection valve to position 5<br>Aspirate 2000 µL at 10 mL/min<br>Wait 3 s	Aspiration of remains of buffer from holding coil and sample, mixing of solution in-syringe
Selection valve to position 8<br>Aspirate 100 µL at 10 mL/min<br>Wait 1 s	Empty remains of sample from the HC into the syringe with air
Relay 1 in OFF	Deactivate stirring

Empty syringe at 0.5 mL/min to head valve position MIDDLE<br>Wait 3 s	Sample loading onto nano-fibers
PORT = 2<br>Call routine „Syringe cleaning" (2x)	Cleaning the syringe 2x with water
Selection valve to position 3<br>Aspirate 25 μL at 1 mL/min from head valve position OUT<br>Wait 3 s	Buffer aspiration
Relay 1 in ON	Activation of stirring
Selection valve to position 6<br>Aspirate 1000 μL at 10 mL/min from head valve position OUT<br>Wait 2 s	Water aspiration for washing
Relay 1 in OFF	Deactivation of stirring
Dispense 2000 μL at 0.75 mL/min to head valve position MIDDLE<br>Wait 3 s	Washing of nanofibers
Injection valve to position INJECT	
Relay 3 in ON for 1 s	Triggering of HPLC
Relay 2 in ON for 1 s	Initiation of Gradient
Wait 300 s	Wait for method restarting

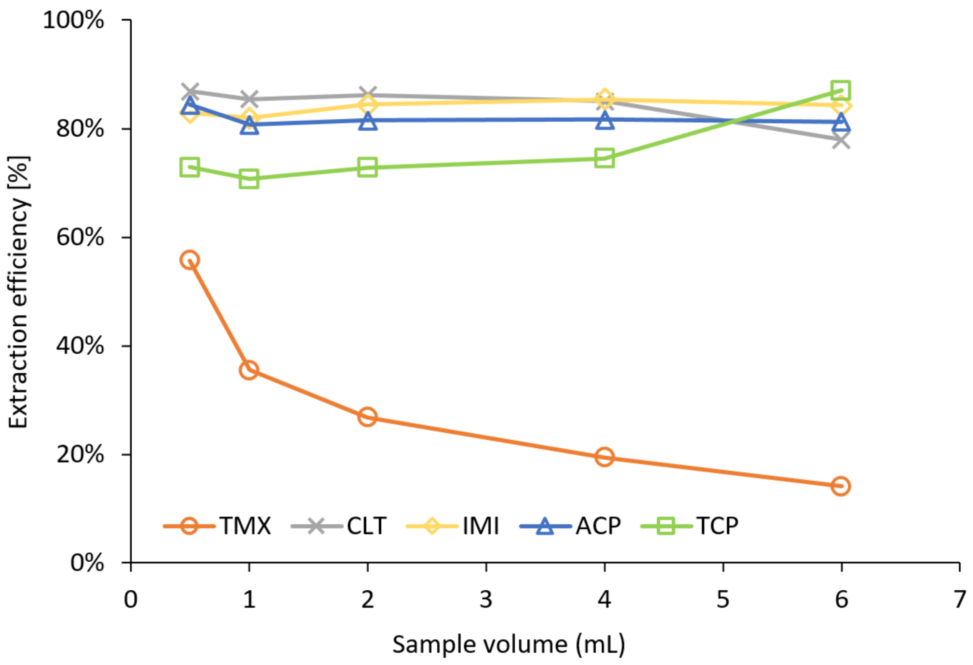

Figure S6. Effect of sample volume on the analytical response. Conditions: 3 layers of PID NF; sample: 1, 2, 4 or 6 mL, 1 mg·L^{-1}, pH adjusted to 3; washing with 1 mL water, elution with 1 mL ACN 100%; off-line measurement, injection volume: 10 μL.

Table S7. Obtained peak characteristics (5 μg· L^{-1}, n = 6).

Analyte	t_R ± RSD%	Tailing factor	R_S	Width (5%) [min]
TMX	7.73 ± 4.59%	1.79	-	0.242
CLT	8.49 ± 6.03%	1.78	4.45	0.235
IMI	8.85 ± 6.13%	1.57	2.14	0.235
ACP	9.34 ± 5.31%	1.41	2.94	0.203
TCP	10.58 ± 2.57%	1.41	8.29	0.192
